# Correlates of perceived social unacceptability of vaping among regular e-cigarette users: a cross-sectional study of a sample of Middle Eastern countries

**DOI:** 10.3389/fpubh.2025.1620863

**Published:** 2025-10-15

**Authors:** Hissa Mohamed AlMuraikhi, Fatima Al Zahraa Chokor, Mohammed Al-Hamdani

**Affiliations:** Department of Public Health, College of Health Sciences, QU Health, Qatar University, Doha, Qatar

**Keywords:** electronic cigarettes, vaping, social unacceptability, pressure by friends, Middle East

## Abstract

**Aim:**

To examine the association between the social unacceptability of vaping and the main correlate, country of residence, as well as other sociodemographic variables in three Middle Eastern countries.

**Methods:**

A cross-sectional online survey study of a convenience sample of regular vapers in Middle Eastern countries was recruited. Recruitment took place through paid advertisements on social media, regular e-cigarette users, completed an online survey. Vapers responded to socio-demographic and social unacceptability questions. Ordinal logistic regression was used for analysis.

**Results:**

*N***=** 428 vapers completed the survey. Male vapers and vapers who currently smoke had lower odds for social unacceptability perceptions relative to females and never smoker counterparts. Vapers in Qatar, experiencing pressure to vape from friends, encountering negative effects, and mod and e-cigarette use was associated with higher odds of social unacceptability perceptions relative to vapers in Egypt, not experiencing pressure from friends, not encountering negative effects, and pod use, respectively.

**Conclusion:**

Relative to vapers in Egypt, those in Qatar reported higher social unacceptability levels, likely due to restrictive legislations, and calls for similar restrictions in Egypt. More awareness is needed to increase social unacceptability among vapers who are males and current smokers. Highlighting potential negative effects from vaping in education campaigns and regulatory restrictions on pod design (compact, sleek, and concealable features) may help increase the social unacceptability of vaping.

## Introduction

Electronic cigarettes are devices that contain batteries to power the device and often contain nicotine in various flavors. A few of the many names that these devices have taken on as their popularity has grown are mods, e-hookahs, and “cig-a-likes” (1). E-cigarettes are currently a major threat to public health worldwide (2), having varying prevalence in different countries. In a study conducted in Qatar, 14% of college students vape (3), According to two Egyptian studies, between 10.6 and 16.5% of participants used e-cigarettes (4). According to Qanash et al. (5), in a sample of Saudi Arabian college students, 27.7% claimed they are currently using e-cigarettes. In the general public, 3.8% of people in the United Arab Emirates, 11.7 to 39.2% of people in Jordan, and 2.2% of people in Saudi Arabia use e-cigarettes (4). Further, in Palestine, 18.1% of a sample of university students reported vaping (6). Although debates about e-cigarettes as a tool for cessation versus a reason for tobacco use initiation continue (7), they are arguably detrimental to public health given the fact that they have been associated with a number of health problems, such as cardiovascular, pulmonary, and developmental effects (8).

Among the United States (US), United Kingdom (UK), Australia, and Canada, Australia has restrictive vaping products legislation, whereby vaping products are only available with a prescription from a doctor and cannot be legally marketed (9, 10). Importing any kind of vaping device into Australia is largely prohibited, although using vapes for the purpose of managing nicotine dependence and quit smoking is legal (11). Canada prohibits vaping products that contain nicotine concentrations above 20 mg/mL (12). According to federal law, to purchase tobacco products, including e-cigarettes, individuals in the United States must be at least 21 years old (13). There are vast differences in e-cigarette regulations in the Middle East (4). For example, in Qatar, the manufacture, importation, marketing, distribution, display, and manufacturing of e-cigarettes is prohibited, while the fact that they are legal for personal use (14). Contrarily, in Jordan, e-cigarette use is legal, and in Iraq, there is no clear law pertaining to e-cigarettes (4). Despite being prohibited by law, e-cigarettes are available for purchase in Egyptian markets (4).

There is evidence that indicates a high level of social acceptability for e-cigarette use—for instance, e-cigarette users consider themselves “committed” to use and not “addicted” (15). However, this social acceptability seems to be true for “social use” but not “daily” use, the latter of which is considered stigmatized by college students (16). Higher vaping identity intensity, the self-identification as a vaper, is associated with lower trust for regulations imposed against vaping by governments (17). When regulations prohibit vaping use, it can still be socially acceptable, in contexts where enforcement is perceived to be weak (18). Studies have even shown sub identities of vapers, “cloud chasers” are the devout vapers with a sense of belonging to a community of vapers, and “substitutes” are ex-tobacco users who aim to escape their previous stigmatized identity and for them vaping is the mechanism to escape their previous identity (19). In young adults, the support for government policy against vaping is far from unanimous, thereby indicating a high level of social acceptability for vaping (20). Holding beliefs that are centered on the idea that vaping is the norm is associated with higher vaping frequency (21, 22) and having strong social connections is associated with increased likelihood of vaping relative to not having any vapers in one’s close social circle (23).

Middle Eastern countries show differences in social acceptability perceptions towards e-cigarettes. For instance: 20.2 to 69% of individuals perceive e-cigarettes as useful aids for quitting smoking (24). According to Jirjees et al. (4), young individuals in Saudi Arabia report using e-cigarettes to quit smoking, while in Jordan, Lebanon, and Qatar, e-cigarettes are perceived as less dangerous than tobacco (3, 4). In Saudi Arabia, 71% of young adults perceived vaping as socially acceptable relative to tobacco smoking (41). In the UAE, e-cigarettes are favored for their variety of flavors and perceived safety (4). In Egypt, vaping is perceived as a fashion statement and a tool for quitting smoking (4).

The Middle East has a wide range of social concerns about e-cigarettes. In the Middle East, 36 to 60.8% of people believe that e-cigarettes are a public issue, with 91.5% acknowledging their harmfulness and 33.5 to 41.8% highlighting their ability to cause nicotine addiction (4, 24). In Saudi Arabia and Egypt, the safety concerns for e-cigarette use are significant (4). People in Egypt also perceive e-cigarettes as ineffective smoking cessation strategies and potentially harmful in the long term. According to Aaiz et al. (25), 32% of participants in Iraq strongly agreed that e-cigarettes are harmful and 92.1% would never encourage their use. In addition, middle school students express more negative feelings about e-cigarettes than traditional cigarettes (25). Younger adolescents often fear the possibility that their parents or another family member will find out they vape, indicating a low level of social acceptance of e-cigarettes (26).

According to Aleyan et al. (9), the degree of laws and regulations in different countries is correlated with the social acceptance of vaping products. Further, the impact of a number of social factors on vapers varies according to their sex and tobacco use status, including peer pressure, exposure to social media content, and product preferences (e.g., type of device used) (23). Although social norms regarding the use of e-cigarettes have been previously explored, social unacceptability (de-normalization) of e-cigarettes is less studied. Evidence suggests the importance of studying social unacceptability, as some users hide their vaping habits in public (26, 27). Therefore, this study aims to examine how regular e-cigarette users perceive the social unacceptability of e-cigarettes and compare three Middle Eastern nations: Qatar, Egypt, and Iraq, while taking into consideration important social and behavioral factors related to vaping. This examinations helps establish whether or not there is a link between the degree of perceived social unacceptability among vapers and the laws currently in place in these nations.

## Methods

### Study design and recruitment

This cross-sectional survey data was collected from February 23 until May 12, 2023 on participants recruited using convenient sampling through paid advertisements on Facebook and Instagram. These advertisements targeted participants who were 18 years old or above and had shown interest in vaping and tobacco products, as determined by the platforms’ algorithms. Ethical approval for the study was obtained from the Research Ethics Board at Qatar University (#QU-IRB 1806-E/23). The participants reviewed an informed consent on the first page of the online survey. To provide consent, participants had to click on “By clicking next, you agree to participate in the survey.” Otherwise, they were unable to proceed.

Sample size calculation for the analysis in this study was performed using G*Power (28). For 15 predictors, a small to medium effect size f^2^ (0.07), power = 0.8, alpha level of 0.05 for a multiple linear regression test, a minimum sample size of N = 282 was needed (but we resorted to ordinal regression due to a violation of multiple regression assumptions).

### Study sample and inclusion/exclusion criteria

The study included participants aged 18–60 years old who were regular vapers (those that use a device once per week or more for the previous 3 months). Additionally, participants were current residents of Arab countries in the Middle East. Individuals were excluded if they were under 18 or more than 60 years old, non-regular vapers, or not a residence of an Arab country. Data from participants who completed data on variables needed for analysis in full were analyzed. If they wished, participants opted in for a prize draw for one $300 gift card.

### Data collection

Eligible social media users who were interested in taking part of this survey were directed to Blue, a survey software offered by Qatar University, to complete the online questionnaire which was a comprehensive cross sectional survey exploring the demographic, vaping behaviors, social aspects, and perceptions of use. Two studies have been published from the same dataset, each examining different aims—exploring correlates of vaping frequency, negative effects and early initiation (29, 30). However, the social unacceptability outcome was used only for the current study. We administered the survey in English and Arabic. The survey was piloted tested and revised for comprehension, length and difficulty prior to use. Participants were offered a prize draw (optional) for a single $300 gift card.

### Measures

Below are the questionnaire variables used and the social unacceptability measure.

#### Questionnaire variables

The questionnaire collected data on various participant characteristics, including age, sex (female, male), country (listing Arab countries), residence status (citizen, resident), and employment status (employed, not employed). Information on tobacco and vaping behavior was also collected such as tobacco use status (ever, never), prior smoking history before vaping (yes, no), vaping frequency per week (1–2, 3–4, and 5–7 days), nicotine content (<20 mg/mL, 20–35 mg/mL, 50–60 mg/mL, do not know), friend pressure to vape (yes, no), exposure to vaping-related ads on social media (yes, no), type of vape device used (mod, pod, e-cigarette, vape pen), flavor preference (yes, no), experience of any negative side effects from vaping (yes, no, do not know), and ever tried to quit vaping (yes, no). These questions have been used in the context of studying other topics in vaping social perceptions and behaviors in the past literature and developed by some of the authors of this study (23, 31).

#### Social unacceptability measure

The survey for this study also included questions on the social unacceptability of vaping that was used for the first time in this study and developed based on guidance from the previous literature [e.g., (16, 19, 23)]. A negative frame was used for social unacceptability in line with viewing vaping as a health issue and placing it in the context of de-normalization. For these questions, participants rated their agreement with five statements: (1) When I vape in public, I worry about the impressions of others about me; (2) When I vape in public, people seem to be bothered by the smell; (3) When I vape in public, people seem to be bothered by the sight of vape clouds; (4) When I vape in public, I feel rejected; and (5) When I vape in the presence of friends who do not vape, they seem bothered. Responses to these items ranged from “strongly disagree” to “strongly agree” on a 5-point Likert scale. The scores for these five items were summed to calculate an overall social unacceptability score and their average was computed by dividing the total score by 5 (number of items). The reliability of the social unacceptability scale was assessed using internal consistency (Cronbach’s alpha = 0.775), and validity was assessed through exploratory factor analysis. We used a Varimax rotation. The exploratory factor analysis confirmed that all items loaded into one factor, explaining 42% of the variance (eigenvalue of factor 1 = 2.66). All items had factor loadings above 0.4. Prior to conducting statistical analysis, we tested for multiple linear regression assumptions and some were violated. We switched to ordinal regression as the analytical approach and collapsed the 5-point scale to a 3-point scale due to small numbers in some levels for the original 5-point scale. Particularly levels 1–2 (strongly disagree to disagree) were changed to 1 (low social unacceptability), 3 (neutral) to 2 (moderate social unacceptability) and 4–5 (agree to strongly agree) to 3 (high social unacceptability).

### Statistical analysis

Sociodemographic and vaping characteristics are summarized as frequencies and proportions and compared across levels of perceived social unacceptability using Pearson’s Chi-square tests. Associations between sociodemographic characteristics and vaping characteristics with perceived social unacceptability levels were initially examined using simple ordinal regression analysis, with perceived social unacceptability level being the dependent variable. A multiple ordinal regression model was used for analysis. To account for confounders in the multivariate analysis, a variable inclusion criterion of *p*-value<0.2 was used based on the *p*-values observed in univariate analysis. The proportional odds assumption was evaluated using the likelihood-ratio test of proportionality and the Brant test, both of which yielded non-significant results, indicating that the assumption was met. Odds ratios (OR) along with 95% confidence intervals were reported. Statistical analyses were completed using Stata, version 18.0.

## Results

Table 1 shows the sociodemographic and vaping characteristics for the total sample (n = 428) as well as by levels of perceived social unacceptability. The majority of participants were males (91%), with 38.3% aged 18–24 years, 38.1% aged 25–36 years, and 34% aged 37 years or older. Most participants were from Egypt (45%), followed by Iraq (33%), and Qatar (12%), with approximately 10% from other Arab countries. A significant proportion were citizens of their residing country (90%) and were employed (60%). Nearly 90% of participants reported being ever-smokers, with 78% indicating they started smoking before vaping. About 85% reported vaping 5–7 days per week, and less than half (47%) reported using vape juice with a nicotine concentration below 20 mg/mL. Friend’s pressure to vape was reported by 15% of participants, and around 48% experienced negative side effects from vaping. Additionally, 41% reported exposure to vaping content on social media ads. Regarding device preference, the majority used pods (35%), followed by mods (34%) and vape pens (16%). Approximately 72% expressed a preference for flavored vape products, and nearly half (48%) reported having attempted to quit vaping at some point.

**Table 1:** Demographics and vaper characteristics, by perceived social unacceptability level (*n* = 428).

Characteristics		Total sample (*n* = 428)	Perceived social unacceptability level			*p*-value*
			Low (*n* = 156)	Moderate (*n* = 190)	High (*n* = 82)	
Age category	18–24 years	164 (38.3)	52 (31.7)	73 (44.5)	39 (23.8)	0.259
	25–36 years	163 (38.1)	65 (39.9)	74 (45.4)	24 (14.7)	
	37–60 years	101 (23.6)	39 (38.6)	43 (42.6)	19 (18.8)	
Sex	Female	37 (8.6)	6 (16.2)	13 (35.1)	18 (48.6)	<0.001
	Male	391 (91.4)	150 (38.4)	177 (45.3)	64 (16.4)	
Country of residence	Egypt	192 (44.9)	86 (44.8)	78 (40.6)	28 (14.6)	0.002
	Iraq	143 (33.4)	46 (32.2)	72 (50.3)	25 (17.5)	
	Qatar	51 (11.9)	11 (21.6)	21 (41.2)	19 (37.2)	
	Others	42 (9.8)	13 (30.9)	19 (45.2)	10 (23.8)	
Residence status	Citizen	383 (89.5)	141 (36.8)	172 (44.9)	70 (18.3)	0.400
	Resident	45 (10.5)	15 (33.3)	18 (40.0)	12 (26.7)	
Employment status	Not employed	131 (30.6)	39 (29.8)	61 (45.6)	31 (23.7)	0.105
	Employed	297 (69.4)	117 (39.4)	129 (43.4)	51 (17.2)	
Tobacco use status	Never smoker	46 (10.7)	8 (17.4)	24 (52.2)	14 (30.4)	0.010
	Ever smoker	382 (89.3)	148 (38.7)	166 (43.5)	68 (17.8)	
Prior smoking history before vaping	No	92 (21.5)	26 (28.3)	42 (45.6)	24 (26.1)	0.077
	Yes	336 (78.5)	130 (38.7)	148 (44.0)	58 (17.3)	
Vaping frequency per week	1–2 days	30 (7.0)	9 (30.0)	11 (36.7)	10 (33.3)	0.167
	3–4 days	31 (7.2)	8 (25.8)	15 (48.4)	8 (25.8)	
	5–7 days	367 (85.7)	139 (37.9)	164 (44.7)	64 (17.4)	
Nicotine content (mg/mL)	<20	201 (47.0)	79 (39.3)	91 (45.3)	31 (15.4)	0.513
	20–35	85 (19.9)	28 (32.9)	37 (43.5)	20 (23.5)	
	50–60	77 (18.9)	30 (39.0)	31 (40.3)	16 (20.8)	
	Do not know	65 (15.1)	19 (29.2)	31 (47.7)	15 (23.1)	
Friend pressure to vape	No	361 (84.4)	138 (38.2)	162 (44.9)	61 (16.9)	0.016
	Yes	67 (15.6)	18 (26.9)	28 (41.8)	21 (31.3)	
Exposure to vaping-related ads on social media	No	252 (58.9)	86 (34.1)	121 (48.0)	45 (17.9)	0.196
	Yes	176 (41.1)	70 (39.8)	69 (39.2)	37 (21.0)	
Type of vape device used	Pod	150 (35.0)	67 (44.7)	62 (41.3)	21 (14.0)	<0.001
	Mod	145 (33.9)	48 (33.1)	77 (53.1)	20 (13.8)	
	e-cigarette	63 (14.7)	21 (33.3)	21 (33.3)	21 (33.3)	
	Vape pen	70 (16.4)	20 (28.6)	30 (42.9)	20 (28.6)	
Flavor preference	No	119 (27.8)	48 (40.3)	53 (44.5)	18 (15.1)	0.353
	Yes	309 (72.2)	108 (34.9)	137 (44.3)	64 (20.7)	
Experienced negative health effects from vaping	No	207 (48.4)	96 (46.4)	84 (40.6)	27 (13.0)	<0.001
	Yes	136 (31.8)	40 (29.4)	63 (46.3)	33 (24.3)	
	Do not know	85 (19.9)	20 (23.5)	43 (50.6)	22 (25.9)	
Ever tried to quit vaping	No	224 (52.3)	87 (38.8)	102 (45.5)	35 (15.6)	0.140
	Yes	204 (47.7)	69 (33.8)	88 (43.1)	47 (23.0)	

Numbers presented are frequencies (percentages).

*p-value obtained using Pearson’s Chi-square tests.

When analyzing vaper characteristics across levels of perceived social unacceptability, significantly higher proportions of females, individuals residing in Qatar, those who had never smoked, those who reported experiencing friend pressure to vape, vapers who primarily used e-cigarettes as their choice of vaping device, and those who encountered negative side effects from vaping were more likely to report higher levels of perceived social unacceptability (*p* < 0.05) (Table 1).

As can be seen in Table 2, male vapers and vapers who are past smokers of traditional cigarettes had lower odds of reporting higher levels of perceived social unacceptability towards vaping relative to female vapers and vapers who never smoked, respectively. Vapers residing in Qatar and vapers who experience pressure from their friends to vape had higher odds of reporting higher levels of perceived social unacceptability towards vaping relative to vapers residing in Egypt, and vapers who do not experience pressure from friends, respectively. Vapers who experienced negative health effects from vaping or do not know if they did we more likely to report higher levels of social unacceptability relative to counterparts that never experienced negative health effects. Finally, vapers who usually use mods or e-cigarettes were more likely to have higher levels of social unacceptability in comparison to those who use pods.

**Table 2:** Associations between sociodemographic and vaping characteristics and perceived social unacceptability level.

Characteristics		AOR (95% CI)
Age category	18–24 years	Reference
	25–36 years	0.8 (0.5, 1.3)
	37–60 years	1.0 (0.6, 1.9)
Sex	Female	Reference
	Male	0.3 (0.1, 0.8)
Country of residence	Egypt	Reference
	Iraq	1.5 (0.9, 2.4)
	Qatar	2.2 (1.1, 4.9)
	Others	1.2 (0.6, 2.4)
Employment status	Not employed	Reference
	Employed	1.0 (0.6, 1.8)
Tobacco use status	Never smoker	Reference
	Ever smoker	0.4 (0.2, 0.9)
Prior smoking history before vaping	No	Reference
	Yes	1.3 (0.7, 2.5)
Vaping frequency per week	1–2 days	Reference
	3–4 days	0.9 (0.3, 2.4)
	5–7 days	0.9 (0.4, 2.0)
Friend pressure to vape	No	Reference
	Yes	1.9 (1.2, 3.4)
Ever tried to quit vaping	No	Reference
	Yes	1.09 (0.7, 1.6)
Experienced negative health effects from vaping	No	Reference
	Yes	1.9 (1.2, 3.0)
	Do not know	2.3 (1.4, 3.8)
Exposure to vaping-related ads on social media	No	Reference
	Yes	0.9 (0.6, 1.3)
Type of vape device used	Pod	Reference
	Mod	1.6 (1.1, 2.6)
	e-cigarette	1.9 (1.1, 3.5)
	Vape pen	1.4 (0.8, 2.6)

AOR, Adjusted Odds Ratio from multiple ordinal regression.

## Discussion

According to Amin et al. (42), earlier studies have mostly concentrated on understanding social unacceptability of non-vapers. This study adds to the literature by providing insights on the correlates of social unacceptability perceptions in the Middle East. This study specifically looked at the relationships between sociodemographic variables and the degree of social unacceptability of vaping among vapers in Egypt, Iraq, and Qatar, three Middle Eastern countries. In our study, vapers from Qatar were more likely than those from Egypt to perceive vaping as socially unacceptable. This likely stems from differences in legislation. Unlike Egypt (32) and Iraq (4, 33), Qatar has strict measures against the production and sale of vaping products (34). In addition, this finding aligns with earlier studies in terms of the connection between the social unacceptability of vaping products and the level of restrictiveness in the country’s legislation (9). The result of this study implies that legislation in this region should be harmonized in order to perhaps lower the social acceptability of vaping in countries like Egypt and Iraq that have less stringent regulations. To fully map the link between the strictness of laws and societal unacceptability, additional research is required to look at other Middle Eastern countries.

In our study, male vapers and vapers with a history of traditional tobacco use were less likely than female vapers and vapers who had never smoked to have high social unacceptability perspectives about vaping. According to earlier research, male tobacco users and vapers are less supportive of tobacco control measures (35). Our study adds to the past findings by showing that these two subgroups also have lower odds of social unacceptability perceptions for vaping. It also suggests that low social unacceptability and low support for policies are aligned. Further, it is aligned with past findings on vaping subcultures which show that “substitutes,” past smokers who are current vapers, use vaping as a way of escaping their stigmatized ex-smoker identity (19). Therefore, it is logical to expect vapers who are ex-smokers to be less likely to appraise vaping negatively from a social standpoint.

Individuals who reported feeling pressured to vape by their peers had higher odds of social unacceptability perceptions towards vaping. According to Groom et al. (26), peer pressure is linked to higher levels of vaping, but it additionally leads to higher odds of vaping being perceived negatively. This unexpected conclusion calls for more research in the future. One possible explanation is that while peer pressure may make vaping easier and less perceived as a behavioral control, it is also seen as an unacceptable behavior that raises feelings of social unacceptability.

Experiencing negative side effects from vaping increases odds of high social unacceptability perceptions in comparison to not experiencing them. This finding complements prior studies that revealed that experiencing negative side effects is one of the key reasons for e-cigarette cessation (36). Based on the literature review, one of the indicators of social unacceptability of e-cigarettes was the harm or the negative health consequences that are associated with it. For example, in Middle Eastern countries e-cigarettes were perceived as harmful (4, 24). Moreover, it was perceived to be harmful in the long term in Egypt (4). Additionally, participants in Iraq strongly agreed that e-cigarettes pose harms (25). Our study suggests that experiencing negative side effects could be related to the fact that they find it less socially acceptable to vape. However, to determine if social unacceptability is the mechanism behind the association between unfavorable side effects and decreased vaping behaviors, further research is necessary. Campaigns that highlight the harmful effects of vaping, however, may contribute to increased social unacceptability of vaping and support for cessation attempts.

In this study users of mods and e-cigarettes higher odds of perceiving high social unacceptability compared to those who used pods. This is logical given that pods are small, discrete and easier to conceal relative to mods (37), which makes mods more visible and therefore related to higher odds of social unacceptability. Further, e-cigarettes are closer in their look to a cigarette stick, and by association to cigarettes, e-cigarettes are associated with higher social unacceptability relative to pods. Our study relates to previous research which has documented a higher proportion of vapers who use pods relative to other products (38), which is consistent with our finding that vapers who use pods have lower odds of social unacceptability relative to other products. Social media interventions may be required to reduce pod social acceptability as they could hinder vaping intentions, especially among young adults (39). Social marketing strategies are required to counteract message that promotes vaping usage, especially in light of social media posts that target younger populations (40).

### Limitations

This study was a cross-sectional and its design does not allow for studying social unacceptability change over time. Social unacceptability may change overtime for different reasons including policies and programs. Future studies should examine social unacceptability differences over time for different countries. Limitations of this study also include the limited range of countries explored, which restricts the generalizability of the results to the Middle East. Future studies should recruit samples that are fully representative of the Middle East. Third, this study was not conducted at a population level to better reflect the true relationship between sociodemographic variables and social unacceptability, further limiting generalizability. Third, while this study examined the relationships between sociodemographic characteristics and social unacceptability, it did not explore the mechanisms that explain these relationships. Future studies should conduct mediation analyses to understand how different sociodemographic variables affect social unacceptability. Further, the social unacceptability scale used in this study was developed based on existing literature rather than through an extensive content generation process. Future research should involve the development of a social unacceptability scale through focus groups of regular vapers and health professionals working in vaping control, prior to testing factor structure. Additionally, our study relied on participants’ recall of their vaping behaviors, which introduces the potential for recall bias. Future prospective cohort study design would mitigate this issue. Finally, further tests of the social unacceptability scale, through confirmatory factor analysis are warranted to better assess it as a measure of the social unacceptability of vaping.

## Conclusion

This study explored differences in levels of social unacceptability across country of residence as well as sociodemographic variables for a vaper sample from Qatar, Iraq, and Egypt. Results revealed an association between each of country of residency, friend pressure to vape, being offered a vaping device, and experiencing negative side effects after vaping with the level of social unacceptability. The study’s implications involve a call for implementing stricter laws in Egypt and Iraq, awareness initiatives about the negative consequences of vaping behavior, and refusal skill training as ways to reduce social unacceptability for using vaping devices.

## Data Availability

The datasets presented in this article are not readily available because of the sensitive nature of the data. The data will only be retained for 5 years from the end date of data collection as this was a condition stated in the informed consent. Requests to access the datasets should be directed to Mohammed Al-Hamdani, email: malhamdani@qu.edu.qa.
